# Minimizing Ionic Losses in DMSO-Free Tin-Based Perovskite Solar Cells

**DOI:** 10.1021/acsenergylett.5c02675

**Published:** 2025-11-14

**Authors:** Paria Forozi Sowmeeh, Shengnan Zuo, Chiara Frasca, Biruk Alebachew Seid, Sercan Ozen, Wentao Liu, Mahmoud Hussein Aldamasy, Yuan Zhang, Fengshuo Zu, Norbert Koch, Martin Stolterfoht, Antonio Abate, Artem Musiienko, Felix Lang

**Affiliations:** † Institute of Physics and Astronomy University of Potsdam, Karl-Liebknecht-Str. 24−25, 14476 Potsdam-Golm, Germany; ‡ 28340 Helmholtz-Zentrum Berlin für Materialien und Energie, 14109 Berlin, Germany; § Department of Physics, Humboldt University of Berlin, 12489 Berlin, Germany; ∥ Electronic Engineering Department, The Chinese University of Hong Kong, Hong Kong SAR, China

## Abstract

Despite the exceptional optoelectronic properties of Pb-based perovskite solar cells, the concerns about their intrinsic instability due to the presence of mobile ions and their potential toxicity are two major obstacles for commercialization. Sn-based perovskites have been revealed as ecofriendly perovskite counterparts. Furthermore, they are believed to exhibit smaller ion-induced instabilities, although thorough investigations are missing. Herein, we investigate the nature of mobile species, quantify the ionic loss within Sn-based perovskite solar cells, and compare with those of Pb-based and mixed PbSn devices. We report over 10-fold lower ion densities with minimal ionic losses in DMSO-free processed Sn samples compared with Pb-based perovskites. The pure Sn-based samples also show the lowest associated ionic losses with sustained device and film stability during prolonged illumination. This study thus propels our understanding of ion migration phenomena in Sn-based devices and paves the way for the development of innovative, stable thin film solar cells with suppressed ion migration.

## Graphical abstract

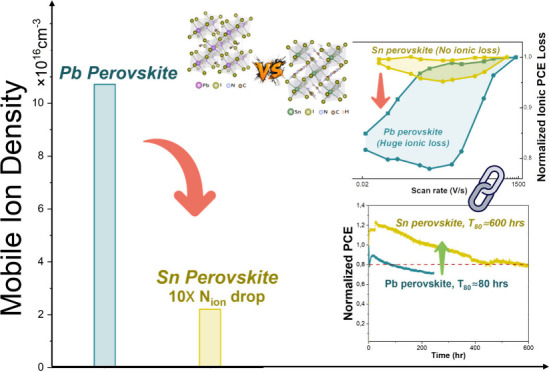

Metal halide perovskites are among the most promising candidates in next-generation photovoltaics owing to the significant progress in terms of their efficiency.^1^ Nowadays, the best performing perovskite solar cells (PSCs) use the benefit of lead as the B-site cation in the ABX_3_ structure of the perovskite.^2^ Nonetheless, toxicity and stability are two major drawbacks impeding PSCs’ progress toward commercialization.^3^ Among all instability origins, presence of mobile ions within the perovskite has been confirmed to be the primary factor, contributing to device degradation^4,5^ as well as healing and metastability.^5–9^ Perovskites are soft semiconductors with loose bonds, making them similar to electrolytes with low activation energy for anions and cations, which in turn, results in facilitated ion migration.^10^ Extensive research has been focused on exploring the ion migration, unrevealing its underlying mechanisms, developing methods to characterize and quantify it, and essentially designing strategies to mitigate it in PSCs. However, despite intensive research, the stability of PSCs remains limited by ion migration if external factors are excluded by proper device encapsulation.^5,11–16^

The most reported range for mobile ion concentrations in lead-based PSCs is 10^17^ to 10^18^ cm^–3^, with activation energies of the most mobile defects between 0.3 and 0.7 eV.^5,17,18^ Yet as summarized in Figure 1a and S1, reported ion densities vary significantly depending on the used measurement technique, perovskite composition, and architecture; hence, caution should be taken when comparing ion density values from different experimental approaches. Moreover, as recently discussed, some electrical measurements cannot quantify ionic densities above, e.g., 10^18^ cm^–3^.^19^ To ensure a fair comparison, the focus of this work is therefore to compare various perovskite compositions by using a consistent measurement technique.

**Figure 1:**
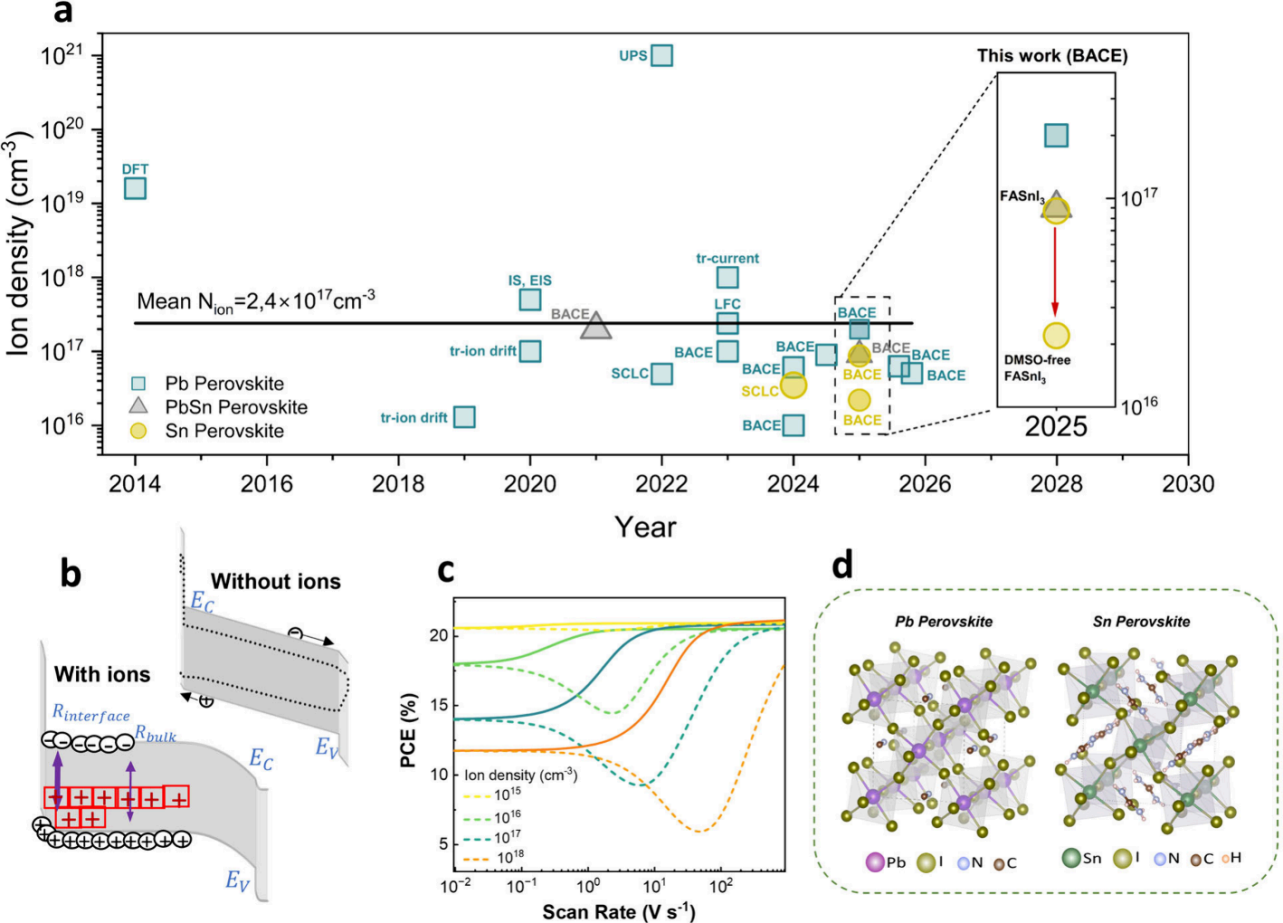
**Ion migration as a source of instability in halide perovskites.** (a) Ion density and the measurement technique used for quantification of mobile ions reported in the literature and this work for Pb-based and Sn-based perovskites.^4,5,16,26–34^ The DMSO-free FASnI_3_ solar cell exhibits almost 10-fold lower mobile ions than Pb-based perovskites. (b) A schematic of the band diagram of the perovskite solar cell showing the accumulation of ions at HTL enhancing bulk and interfacial recombination. (c) Effect of mobile ion concentration on solar cell performance. Figure 1b and c are color-modified and reproduced or adapted from [^5^]. Available under a CC-BY 4.0 Copyright © 2024 Springer Nature. (d) Crystal structures of Pb-based and Sn-based perovskites.

Under operation, ion build-up screens the built-in electric field, expands flat-band regions, and promotes charge accumulation at the hole-selective layer (HTL), enhancing both interfacial and bulk recombination (Figure 1b), as demonstrated by Thiesbrummel et al.^5^ Their theoretical modeling revealed that the ionic losses and ion-related instability diminish when the mobile ion concentration is reduced to below 10^15^ cm^–3^ (Figure 1c).

To reduce the impact of ionic field screening, Zhang et al. introduced a starch-polyiodide supermolecule buffer layer in PSC. This layer promotes stability of the device by suppressing the ion migration, lowering ion concentration from 1.7 × 10^17^ cm^–3^ to 3.3 × 10^16^ cm^–3^.^20^ In parallel, extensive efforts have been devoted to surface passivation in an attempt to mitigate ion migration,^21–24^ which, however, do not address the root cause of the ion migration present. Despite advancements in understanding ion migration in Pb-based perovskites, achieving full control over this process remains an unresolved challenge, limiting the long-term stability of devices, which have yet to consistently achieve stability beyond one year.^5,25^

In the competition of overcoming the toxicity of lead-based perovskites, tin, as a relatively low-toxicity ion,^35^ with high carrier mobility^36^ and low exciton binding energy, is expected to show excellent optoelectronic properties.^3^ Nowadays, remarkable device performance has been achieved by Sn-based perovskites, with power conversion efficiencies (PCEs) pushed up to 17.13%^37^ and stabilities demonstrating over 1300 h under MPP tracking, maintaining 96% of the initial PCE.^38^ However, despite the exceptional potential of tin in reaching stable and efficient devices, only limited theoretical studies have been conducted on the ion dynamics in Sn-based PSCs.

Figure 1d illustrates the crystal structures of Pb-based and Sn-based perovskites, highlighting their potentially distinct defect chemistry due to the substitution of Pb^2+^ with Sn^2+^. Interestingly, theoretical predictions, e.g.,^48^ by Ighodalo et al.,^39^ reported an increased ion migration activation energy (0.85 eV) in CsSn­(I_0.4_Br_0.6_)_3_. They attributed it to the strong bond between Sn and halides, which results in increased activation energy (*E*_*a*_) for mobile ions. Dey et al.^40^ reported suppressed ion migration through atomistic ab initio simulations in PbSn and Sn-based perovskite due to higher activation energy for ionic transport as a result of substituting Pb with Sn. Conversely, Thiesbrummel et al., demonstrated clear evidence of ion migration in mixed PbSn PSCs.^4^ Notably, ion migration has never been studied in FASnI_3_, the most relevant composition for the lead-free perovskite field, due to its perfect solubility and potential efficiency.^41,42^

In this work, we unexpectedly find that FASnI_3_ solar cells prepared using a DMSO-free solvent exhibit the lowest density of mobile ions and ion-associated losses lower than Pb-based counterparts, with sustained device stability under prolonged operational conditions. These results open new pathways for developing stable solar cells with suppressed ionic losses and reduced degradation.

To systematically study the ion dynamics in perovskite devices, we investigated the most widely used PSCs. Details of perovskite compositions, the device structure investigated in this study, and corresponding PCEs are presented in Table 1. These compositions were selected as they represent the most widely used in the PSC field, offering benchmarks for performance and ion migration characteristics. Triple-cation perovskite, with a Cs_0.05_(MA_0.02_FA_0.98_)_0.95_Pb­(I_0.98_Br_0.02_)_3_ perovskite composition (denoted as ‘*CsMAFAPbI*_*3*_’ throughout the paper) and a champion PCE of 21.5%, represents the candidate for Pb-based devices. As for assessing the ion dynamics of tin incorporated PSCs, a mixed Pb–Sn (*‘CsMAFA­(PbSn)­I*_*3*_ *’*) device with a (Cs_0.1_MA_0.3_FA_0.6_)­(Pb_0.5_Sn_0.5_)­I_3_ perovskite composition and 14.2% PCE was studied, which contains a mixture of Pb and Sn in a 50:50 ratio as the B-site cations. Finally, aimed at the ion migration dynamics on Sn-only solar cells, a composition of FA_0.87_PEA_0.13_SnI_3_ perovskite (referred to as *‘FASnI*_*3*_ *’*) was employed. Typically, these are processed using dimethyl sulfoxide (DMSO) as the solvent, and, in our case, we reach a PCE of 6.8%. To explore an alternative to traditional DMSO-based processing, which can oxidize tin and remain trapped within the perovskite bulk due to strong bonding,^43^ we used a DMSO-free solvent system. This approach represents an emerging direction in Pb-free perovskite research, where engineered solvent and antisolvent combinations aim to reduce the likelihood of Sn oxidation and solvent entrapment, enhancing the stability and device performance. The (*‘DMSO-free FASnI*_*3*_ *’*) was proccessed utilizing a mixture of dimethylformamide (DMF) and 1,3-dimethyl-2-imidazolidinone (DMI) reaching 5.6% of PCE. A detailed description of fabrication process of the solar cells can be found in the Supporting Information (SI).

**Table 1:** Perovskite Solar Cell Architecture with Corresponding Perovskite Compositions, Corresponding PCEs, and Bandgaps

Perovskite composition	Structure	PCE (%)	Bandgap (eV)
CsMAFAPbI_3_	ITO/MeO-2Pacz/Pb-perovskite/C_60_/BCP/Cu	21.5	1.56
CsMAFA(PbSn)I_3_	ITO/PEDOT: PSS/PbSn-perovskite/C_60_/BCP/Cu	14.2	1.25
FASnI_3_	ITO/PEDOT: PSS/Sn-perovskite/C_60_/BCP/Ag	6.8	1.40
DMSO-free FASnI_3_	ITO/PEDOT: PSS/Sn-perovskite/C_60_/BCP/Ag	5.6	1.39

We initiated our study by analyzing stabilized (0.5 Hz) current density–voltage (J-V) characteristics of all compositions (Figure 2a). In Pb-based perovskites, typically, forward scan, i.e., from 0 to 1.2 V, exhibits a lower fill factor (FF)_forward_ = 0.68 compared to reverse scans from 1.2 to 0 V where FF_reverse_ = 0.76. Interestingly the J-V results reveal inverted hysteresis in devices incorporating Sn in their perovskite compositions (FF_reverse_ = 0.59 compared to F _forward_ = 0.60), though we note that inverted hysteresis appears in Pb-based compositions too at higher scan rates^44^ (Figure S2, **SI**). Various theories have been hypothesized as the potential cause for inverted hysteresis such as barriers for efficient charge extraction,^45^ whether originating from a specific perovskite composition, device structure, processing procedure, or ion accumulation at perovskite interfaces.^46^ Of all possible reasons, ionic defect redistribution at interfaces of perovskite with charge transport layers and the resultant Fermi level shift is fully consistent with tunable inverted hysteresis.^35^

**Figure 2:**
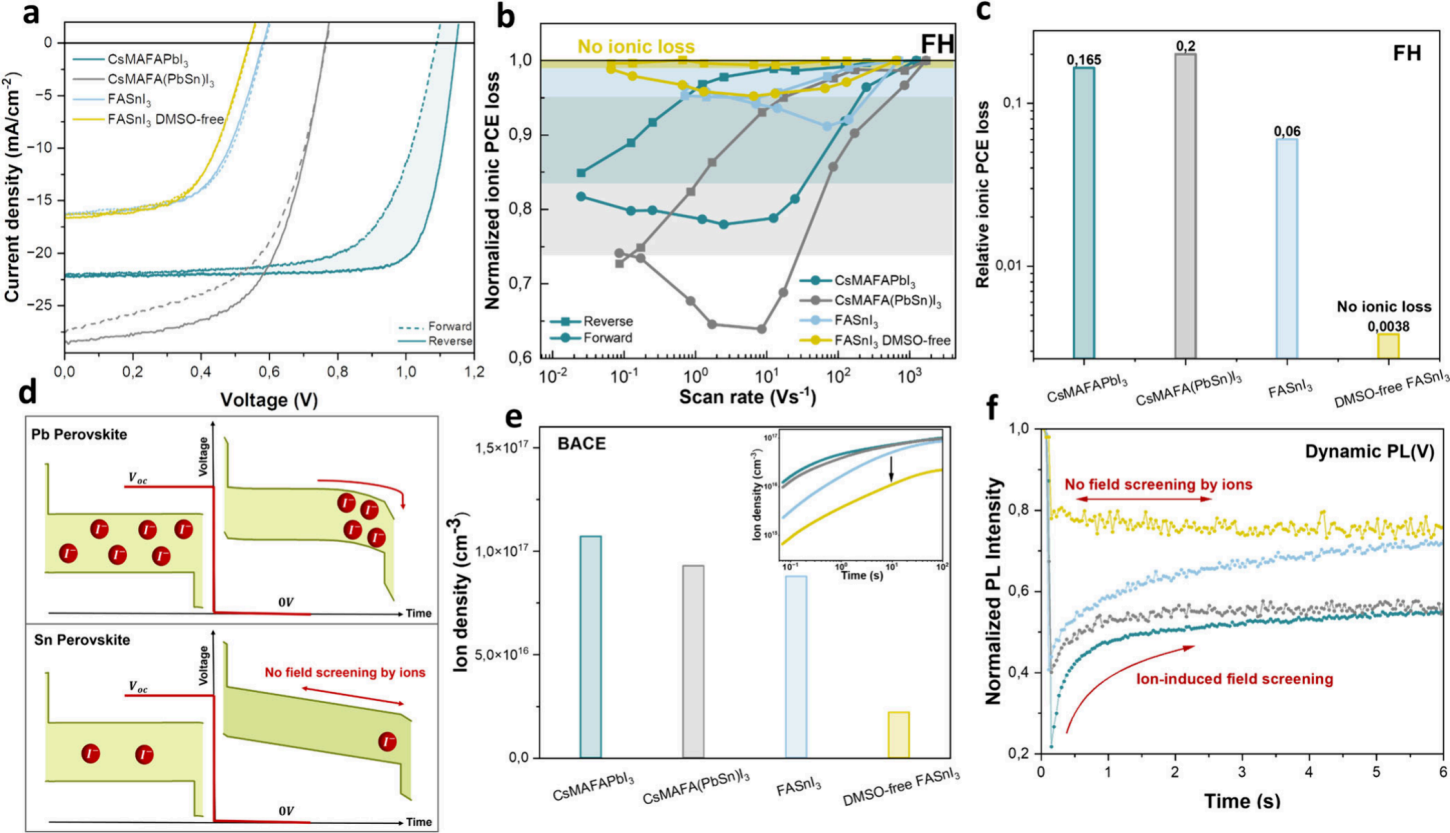
**Ion dynamics in Pb and Sn-based perovskite solar cells.** (a) Current density–voltage characteristics of perovskite solar cells with different perovskite compositions (at 0.5 Hz and 25 °C) measured in the forward and reverse directions. (b) The normalized ionic PCE from fast hysteresis measurement for the different perovskite solar cell and ionic loss as a result of ion dynamics within the device. DMSO-free FASnI_3_ solar cells exhibit no ionic loss. (c) Relative ionic PCE loss reported for different perovskite compositions. d) A schematic of ionic transport during bias-assisted charge extraction (BACE) of the perovskite solar cell for Pb and Sn perovskite. (e) Measured ion density upon switching from *V*_oc_ to *J*_sc_ with BACE and the resultant ion density as the inset, and (f) extracted peak heights from voltage-dependent photoluminescence measurement of each perovskite solar cell upon switching from *V*_oc_ to *J*_sc._.

To quantify PCE loss from ionic transport, we carried out fast hysteresis (FH) measurements, introduced by Le Corre et al.,^14^ a methodology in which ionic loss is estimated via conducting J-V measurements within a wide range of scan rates. In this context, the steady-state PCE refers to the point where reverse (■) and forward (●) curves converge at slow scan speeds, whereas ion-freeze PCE is the convergence point at fast scan speeds where the electric field variation outpaces ionic diffusion rate; hence, ions are effectively immobilized. The difference between PCE_steady‑state_ and PCE_ion‑freeze_ corresponds to the ionic loss within the system.

The PCEs of the Pb and Sn-based solar cells adapted from FH measurement and the resultant ionic losses are demonstrated in Figure S3. Figure 2b and 2c represent the normalized ionic PCE loss (PCE/PCE_max_), and the resultant relative ionic PCE loss (PCE_ion‑freeze_ – PCE_steady‑state_)/PCE_max_ for each perovskite composition, respectively. FH measurement yields a relative ionic loss of approximately 0.165 for CsMAFAPbI_3_ and 0.2 for CsMAFA­(PbSn)­I_3_ devices. In contrast, for the DMSO-processed FASnI_3_ device, a low ionic loss of approximately 0.06 was observed. In the meantime, we observed essentially no ionic loss (∼0.003) for the DMSO-free FASnI_3_ solar cell, suggesting that the impact of mobile ions is substantially mitigated.

The FH-measured hysteresis peak positions (see Figure 2b and Figure S4) reflect the transient time of ions through the active layer, serving as an indicator of ionic mobility. The occurrence of peak hysteresis at higher scan rates in FASnI_3_ highlights the presence of fast mobile ions. At slow scan rates, these ions have sufficient time to accumulate at the interfaces, which creates a localized electric field opposing the charge flow, resulting in inverted hysteresis. In contrast, Pb-based devices, such as CsMAFAPbI_3_ and CsMAFA­(PbSn)­I_3_, exhibit slower ion migration. At fast scan rates, these slow-moving ions cannot redistribute effectively, impeding charge transport and inverted hysteresis.

To assess the mobile ion density in Pb and Sn-based PSCs, we then conducted bias-assisted charge extraction (BACE) measurement.^47^ For this purpose, the solar cell was kept under *V*_oc_ in the dark for a sufficient duration, allowing ions to redistribute evenly throughout the perovskite. Subsequently, the condition was abruptly switched to 0 V, causing the ions to drift to perovskite interfaces with charge transport layers, as illustrated in Figure 2d. The density of mobile ions is then estimated by integrating the external current (seeFigure [Fig fig2]e).^14^ Based on the BACE measurement, we report ionic densities exceeding 10^17^ cm^–3^ for CsMAFAPbI_3_ and 9 × 10^16^ cm^–3^ in CsMAFA­(PbSn). On the other hand, lead-free FASnI_3_ device contains lower ion densities of 8.7 × 10^16^ cm^–3^. Surprisingly, DMSO-free FASnI_3_ devices distinctly exhibit lower ion density, with values decreasing significantly to 2.2 × 10^16^.

Another approach introduced by Thiesbrummel et al.^4,48^ utilizes voltage-dependent photoluminescence transient measurement (dynamic PL­(V)) to independently verify the impact of mobile ions on field-screening. Building on these findings, we aimed to investigate evidence of ion dynamics in CsMAFAPbI_3_, FASnI_3_, and DMSO-free FASnI_3_ solar cells using a dynamic PL­(V) technique. For this purpose, we monitored the emitted PL over time while switching from *V*_oc_ (open circuit voltage) to *J*_sc_ (0 V). The evolution of PL­(V) over time is illustrated in Figure S5a-d, with Figure 2f further depicting the corresponding PL peak intensities as a function of time. Initially, at *V*_OC_ conditions, mobile ions are homogeneously distributed, and radiative recombination is dominating. Upon switching to *J*_SC_, PL is quenched due to the rapid extraction of charges, facilitated by mobile ions. In particular, CsMAFAPbI_3_-based devices, which exhibit higher fill factors, show rapid PL­(V) quenching due to efficient charge extraction and superior charge transport properties.^49^ Nevertheless, under the *J*_sc_ condition, mobile ions start accumulating at perovskite interfaces, which in turn results in screening of the internal electric field. Hence, the observed PL development is a consequence of less efficient charge extraction over time.^4,50,51^ This dynamic behavior is particularly evident in CsMAFAPbI_3_, CsMAFA­(PbSn)­I_3_, and FASnI_3_ devices displaying a clear PL quench followed by development trend, indicative of ionic redistribution influencing charge collection. By contrast, the DMSO-free FASnI_3_ does not exhibit such a behavior implying suppressed ion migration.

To gain a detailed insight of the ionic species in lead and tin PSCs, we extended our characterization to temperature-dependent fast hysteresis (FH­(T)) from 25 °C to 45 °C. Ion migration is classified as a thermally activated process, and thus, we expect faster ionic motion upon increasing temperature. This temperature range was intentionally chosen as it represents realistic operating conditions where mobile ions remain active without inducing thermal degradation, ensuring reliable and meaningful extraction of activation energies. As it is shown in Figure S6, we observe an increase in peak scan rate, hysteresis index, and a shift in peak hysteresis scan rate in accordance with faster ionic motion at elevated temperatures.^27,28^

In an effort to further distinguish between the ionic species in different perovskite compositions, we recorded FH­(T) on CsMAFAPbI_3_, CsMAFA­(PbSn)­I_3_, FASnI_3_, and DMSO-free FASnI_3_ devices and then extracted the peak hysteresis scan rates (Figure 3a). Plotting these results over 1/*T* then allows extraction of the activation energies (*E*_*a*_) utilizing Arrhenius law $\nu_{d}=\nu_{0}\exp\left(-\frac{E_{a}}{K_{B}T}\right)$ , see Figure 3b. The CsMAFAPbI_3_ device exhibits the activation energy of 0.54 eV, which lies within the previously reported values for mobile iodide species.^12,53–55^ The CsMAFA­(PbSn)­I_3_ device demonstrates slightly smaller activation energy of 0.49 eV compared to the pure Pb-composition. This reduction can be attributed to incorporation of Sn^2+^, which is prone to oxidize via Sn^2+^ → Sn^4+^ + 2e^–^ to Sn^4+^, leaving Sn^2+^ vacancies^56^ (*V*_Sn_). The presence of V_Sn_ facilitates the iodide migration, resulting in a smaller activation energy for iodide.

**Figure 3:**
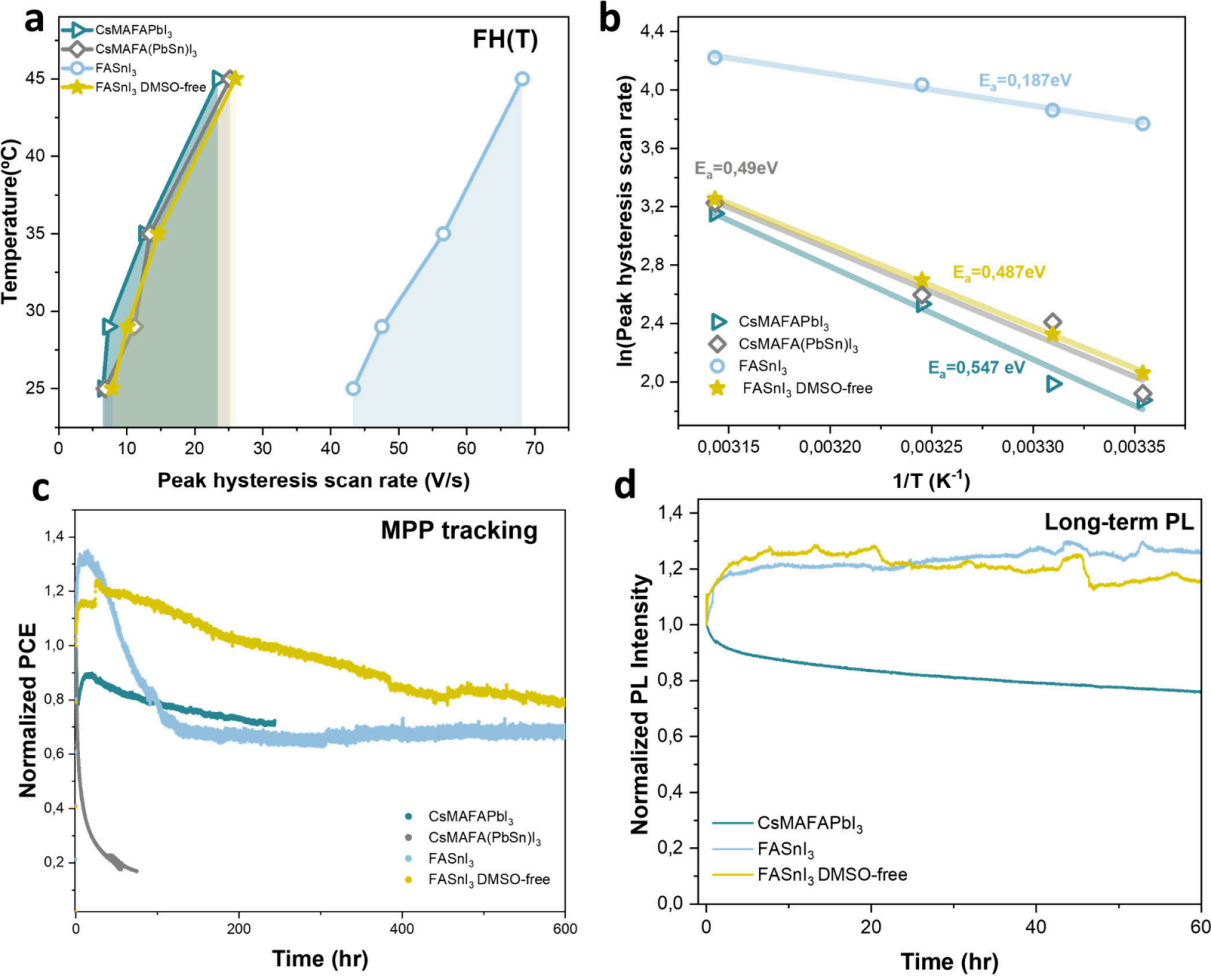
**Temperature-dependent ion dynamics in perovskite solar cells and PL stability of Sn and Pb perovskite films.** (a) FH­(T) results for different perovskite compositions from 25 to 45 °C according to the corresponding scan rate. Peak scan rate corresponds to the minimum of the difference in forward and reverse scans in the PCE FH curve. (a) Peak scan rate hysteresis adopted from FH­(T). (b) The corresponding activation energies calculated from Arrhenius law. (c) MPP tracking of the CsMAFAPbI_3_, CsMAFA­(PbSn)­I_3_, FASnI_3_, and DMSO-free FASnI_3_ devices under 1 sun illumination. The slow degradation for the DMSO-free device under MPP tracking indicates that DMSO removal is a promising way for stable Sn devices. (d) Normalized PL intensity (i.e., PL­(*t*)/PL­(0)) for Pb vs Sn-based perovskite films.

In the meantime, the FASnI_3_ device shows significantly lower activation energy of 0.18 eV compared to its mixed (PbSn) counterpart. While the increased V_Sn_ defect density as a result of intrinsic instability of Sn^2+^ contributes to this effect, additional factors also play a major role. A faster ionic species (Figure S3) and higher temperature-dependent mobility can be understood from the lower Goldschmidt tolerance factor, contributing to a more expanded lattice of the FASnI_3_ perovskite, which facilitates iodide migration.

Then again, an increased activation energy of 0.48 eV was calculated for the DMSO-free FASnI_3_ device. DMSO, a dipolar aprotic solvent, serves as an oxidizing agent for Sn^2+^, promoting higher density of Sn^4+^ ^57^ (refer to SI for details regarding XPS, also see Figure S7), along with a Sn^2+^ vacancy. Density functional theory (DFT) calculations^58^ have shown that the presence of a V_Sn_ defect in FASnI_3_ perovskite initiates an I-rich environment, paving the way for iodide migration. Absence of DMSO as an oxidizing environment (DMSO-free FASnI_3_ device), thus should lower the density of V_Sn_ and minimize iodine migration. Thus, high activation energy and low ionic density in DMSO-free FASnI_3_ perovskite suggests that the V_Sn_ defect dominates the ionic mobility, albeit being low in number. Given the large migration barrier for V_Sn_, the existence of this ionic species does not contribute to the ionic loss of the device.

To corroborate our conclusions, we tested device operational stability by maximum power point (MPP) tracking (Figure 3c, for more details, refer to SI). As exhibited in Figure 3c, the CsMAFA­(PbSn)­I_3_ shows the lowest stability (*T*_80_ < 1 h) which correlates with its high relative ionic PCE loss (0.2%, Figure 2c), whereas CsMAFAPbI_3_ exhibits T_80_ > 80 h with comparatively small ionic loss (0.165).

As for pure Sn-based samples, we analyzed the stability by averaging 8 cells of DMSO and DMSO-free devices. While the FASnI_3_ device shows *T*_80_ ∼ 90 h with 0.06 relative ionic PCE loss, we can clearly see a slow degradation for DMSO-free devices under MPP tracking (*T*_80_ ∼ 600 h), indicating that DMSO removal is a promising way for stable Sn devices, which is consistent with lower ion density and negligible relative ionic PCE loss (0.0038).

The long-term photoluminescence (PL) stability of CsMAFAPbI_3_, FASnI_3_, and DMSO-free FASnI_3_ films under 1 sun illumination is presented in Figure 3d. In Pb-based devices, photoinduced trap formation promotes nonradiative recombination, leading to a gradual deterioration of PL over time. In contrast, the Sn-based perovskite films demonstrate improved stability, which can be attributed to the intrinsically lower ion density and reduced ionic loss in these compositions. Furthermore, Sn-based perovskite films are highly susceptible to the oxidation of Sn^2+^ to Sn^4+^, which initially creates trap states. Upon illumination, however, some of these traps can be partially passivated by the accumulated Sn^4+^ species, resulting in a temporary increase in the PL intensity. DMSO is known as a coordinating solvent that forms an intermediate complex during crystallization, thereby decreasing the initial defect density and slowing Sn^2+^ oxidation. The observed PL enhancement in Sn-based perovskites therefore does not indicate superior intrinsic stability but rather reflects a dynamic balance between trap formation and passivation. Consequently, the long-term PL stability of DMSO-free FASnI_3_ films is inferior to that of DMSO-processed films, since the absence of coordinating solvents accelerates crystallization and facilitates oxidation, leading to faster degradation. Thus, the observed PL evolution is primarily governed by chemical degradation (Sn^2+^ oxidation and defect dynamics) rather than by ion migration alone. These results highlight the inherent stability of Sn-based perovskites, emphasizing the importance of optimizing contact interfaces to fully realize stable and efficient tin-based PSCs in future developments.

In summary, we report on the nature of ion migration in both Pb- and Sn-based perovskites. Ion migration presents a significant challenge in metal halide perovskites, contributing to device instability and degradation, despite notable advancements in efficiency. This issue remains unresolved, limiting the broad application of this technology for long-term and reliable use in commercial photovoltaics. We studied mobile ions in the most relevant perovskite compositions in the field. These compositions contain pure Pb-based, mixed Pb–Sn, pure Sn-based on the DMSO solvent, and pure Sn-based on the DMF-DMI solvent. The Pb-based PSC contains the highest ion densities exceeding 10^17^ cm^–3^. Incorporation of Sn in Pb–Sn mixed perovskite slightly reduces ion density to 9 × 10^16^ cm^–3^. Sn-based perovskites using DMSO solvent illustrate a lower density of ions (8.7 × 10^16^ cm^–3^) while Sn-based perovskites utilizing DMF-DMI solvent exhibit the lowest ion density of 2.2 × 10^16^ cm^–3^, which is almost 10-fold lower than that observed in Pb-based perovskites. The Sn-based samples show minimal ionic losses and maintain excellent device stability over time with *T*_80_ ∼ 600 h.

DMF-based Sn perovskites show the lowest ion density, opening pathways for stable thin-film solar cells with ease of processing from solution. We strongly believe that by further improving the technology and reducing defect density in Sn-based perovskites, the ion concentration can be reduced even further.

## Supplementary Material



## Data Availability

The data that support the findings of this study are available from the corresponding author upon reasonable request.

## Notes

Full details of experimental procedures can be found in the Supporting Information. Experimental procedures for solar cell fabrication, additional supplementary data and characterization regarding the impact of ion dynamics in different perovskite compositions, reported activation energies in literature and this work for ion migration, JV characteristics of CsMAFAPbI_3_ and CsMAFA­(PbSn)­I_3_ at fast scan rates indicating inverted hysteresis, PCEs, ionic PCE loss, and peak hysteresis scan rates obtained from FH measurement for perovskite compositions under investigation, the PL evolution in terms of wavelength upon switching from *V*_oc_ to 0 V, PCEs adopted from temperature-dependent fast hysteresis (T-FH) of investigated perovskite compositions from 25 °C to 45 °C, and XPS data for quantifying Sn^4+^ (PDF)
