# Supplementary material for: Minimizing Ionic Losses in DMSO-Free Tin-Based Perovskite Solar Cells
Source: ACS Energy Lett. 2025 Nov 14;10(12):6334–41. doi: 10.1021/acsenergylett.5c02675 (PMC12706833; doi:10.1021/acsenergylett.5c02675)
Supplement: Supplementary file 1 [file nz5c02675_si_001.pdf]

Supplementary Information

Minimizing Ionic losses in DMSO-free Tin-based Perovskite Solar Cells

Paria Forozi Sowmeeh [a,ǂ], *Shengnan Zuo* [b,ǂ], *Chiara Frasca* [b], *Biruk Alebachew Seid* [a], *Sercan Ozen* [a], *Wentao Liu* [b], *Mahmoud Hussein Aldamasy* [b], *Yuan Zhang* [c], *Fengshuo Zu* [b,c], *Norbert Koch* [b,c], *Martin Stolterfoht* [d], *Antonio Abate* [b]*, *Artem Musiienko* [b]*, *Felix Lang* [a]*

[a] Institute of Physics and Astronomy University of Potsdam Karl-Liebknecht-Str. 24–25, 14476 Potsdam-Golm, Germany

[b] Helmholtz-Zentrum Berlin für Materialien und Energie, 14109, Berlin, Germany

[c] Department of Physics, Humboldt University of Berlin, 12489 Berlin, Germany

[d] Electronic Engineering Department, The Chinese University of Hong Kong, Hong Kong SAR, China

*Corresponding Authors: Felix Lang felix.lang1@uni-potsdam.de, Artem Mussienko artem.musiienko@helmholtz-berlin.de , Antonio Abate antonio.abate@helmholtz-berlin.de

ǂThese authors contributed equally.

Experimental Procedures

Perovskite *$CsMAFAPbI_3$* Solar Cell Fabrication*:*

Substrate and HTL preparation: pre-patterned glass/ITO substrates were sonicated for 2 minutes in Acetone, followed by 2 minutes in 3% Hellmanex solution in deionized (DI) water, 2×2 minutes in DI water, 10 minutes in Acetone and finally 10 minutes in isopropanol (IPA). After sonication, the substrates were dried with a nitrogen gun and cleaned via UV-ozone cleaner for 30 minutes before transferring to the N_2-filled glovebox.

As for HTL layer, 0.002g MeO-2Pacz was dissolved in 1ml Ethanol and sonicated until a clear solution was achieved.

Perovskite precursor: 0.0036g MABr, 0.0181g MACl, 0.2248g CsI, 0.27698G FAI, and 0.9091g PbI_2 (with 16% excess PbI_2) weighed and dissolved in 1ml of a DMF:DMSO mixture with 5:1 ratio. The solution was stirred for 4 hours in N_2-filled glovebox at 45°C.

Device Fabrication: 120µL of HTL was dynamically spin-coated on the ITO-patterned substrates at 3000 rpm for 30 s, prior to annealing the samples for 10 minutes at 100°C. After the samples cooled down, 90 µL of perovskite solution was statically spin-coated on top within 2 steps: 10 s at 1000 rpm and a ramp of 334 rpm/s which was followed by 40 s at 5000 rpm at a ramp of 2000 rpm/s. 250 µL of chlorobenzene (CB) was dripped into the sample 12 s before the end of spin coating. Finally, substrates were annealed for 20 minutes at 110°C. The whole process took place inside a N_2-filled glovebox.

After annealing the samples were transferred to the evaporator where C60 (30 nm), 2,9-Dimethyl-4,7-diphenyl-1,10-phenanthroline BCP (8 nm) and copper (100 nm) were deposited under vacuum. Finally, the active area of 0.06 cm^2 was achieved.

Perovskite *$CsMAFA(PbSn)I_3$* Solar Cell Fabrication*:*

Substrate and HTL preparation: Pre-patterned ITO/glass substrates were subjected to plasma cleaning following the initial cleaning process previously described. Then, filtered PEDOT: PSS via 0.45 µL PVDF, was spin-coated on the samples for 10s at 500 rpm followed by 30s at 4000 rpm. Samples were annealed at 140 °C for 20 minutes.

Perovskite precursor: 0.09353 g CsI, 0.371.46 g FAL, 0.1716 g MAI, 0.0282 g SnF_2, 0.0084g GuaSCN, 0.008 g GIHCL, 0.82982 g PbI_2, and 0.670 g SnI_2 were stirred for 40 minutes at 45°C prior to adding Sn powder and stirring for 10 minutes. The final solution was filtered via 0.2 µm PTFE filter.

Device Fabrication: The perovskite solution was spin-coated at 1000 rpm with an acceleration of 200 rpm/s for 10 seconds, followed by 4000 rpm with an acceleration of 1000 rpm/s for 40 seconds. at last 20 s, 250 µL of CB was dripped on to the samples and the samples were anneal for 10 minutes at 100 °C and 10 minutes at 65 °C. Consequently, 1 mg of Ethylenediammonium diiodide ($EDAI_2$) was dissolved in 1mL isopropanol and 1mL toluene and stirred for 3 hours at 70 °C. the $EDAI_2$ was spin-coated at 4000rpm with 1333 acceleration, prior to annealing for 5 minutes at 100 °C.

Finally, C60 (30 nm), 2,9-Dimethyl-4,7-diphenyl-1,10-phenanthroline BCP (8 nm) and copper (100 nm) were deposited under vacuum. Finally, the active area of 0.06 cm^2 was achieved.

Perovskite $FASnI_3$ and DMSO-free $FASnI_3$ Solar Cell Fabrication*:*

Substrate and HTL preparation*:* Patterned ITO glasses were sonicated by using 2 % Mucasol/water solution, DI water, Acetone and IPA for 15 min at 40°C sequentially. ITO substrates were cleaned in UV-ozone cleaner for 15 min. Then, 150 uL PEDOT: PSS dispersion was apin-coated at 4000 rpm for 40 s on top of glass/ITO, followed by annealing at 140°C for 30min in the air. Noted that PEDOT: PSS dispersion was filtered by a 0.45 µm filter before use. Afterwards, glass/ITO/PEDOT: PSS was transferred into N_2 glovebox for perovskite deposition.

Perovskite precursor: For SnI_3 device, 0.172g of FAI and 0.372g of SnI_2 were dissolved in 1mL DMSO forming a nominal concentration of 1M, followed by adding 13mol% PEAI and 10mol% SnF_2 as additives, and mixed in a shaker at room-temperature.

For DMSO-free SnI_3, FAI 0.206g and SnI_2 0.446g at a concentration of 1.2M in DMF:DMI (6:1/v:v), was followed by adding 13mol% PEAI and 10mol% SnF_2 as additives. The solution was stirred in a shaker for two hours at room-temperature, then added 0.5V tBP to a volume V of the perovskite solution in a new vial and mixed.

For DMSO solvent system, 100 µL perovskite solution was spin-coated at 5000 rpm for 60 s with a ramp of 1000 rpm/s, 150 µL CB as anti-solvent was dripped onto samples at 40 s after spin coating, prior to annealing on a hotplate for 10 minutes at 100°C.

For DMF:DMI solvents system, 100 µL perovskite solution was spin-coated at 5000 rpm for 50 s with a ramp of 1000 rpm/s, 100 µL p-xylene was used as anti-solvent after 15 s. Finally, the substrate was annealed on a hotplate at 100°C for 10 min.

Note that the tin perovskite precursor inks were filtered by a 0.2 µm PTFE filter before use in the N_2 glovebox.

Device fabrication: Finally, 23 nm C60, 8 nm BCP and 100 nm Ag were evaporated on the active area 0.16 cm^2.

Device Characterizations

Fast Hysteresis (FH) Measurements: Fast J–V curves were acquired by applying a triangular voltage pulse to the solar cells, starting near the open-circuit voltage (Voc). The pulse included a reverse sweep from Voc to −0.1 V, followed by a forward sweep back to Voc, at various frequencies or scan rates ($V\ s^{-1}$), using the FastChar setup provided by FastChar UG. The holding time at Voc was set to be five times the total sweep duration. The cell's voltage response was recorded with an oscilloscope, using an external load resistance of ≤10 Ω. Although the measurement hardware and protocols differed from standard J–V (Keithley-based) setups, J-V curves extracted from FH measurement at slow scan rate (0.5Hz) matched those obtained from the standard JV setup. As for T(FH) the temperature of the device under investigation was set to desired value using a temperature controller.

BACE Measurements: For dark BACE measurements, the device was initially biased near its open-circuit voltage, where the injected charge balances the short-circuit current. After a defined delay period—typically about five times longer than the observed charge extraction time under collection bias, a 0 V bias was applied to extract both the injected and capacitive charges. This delay allowed ionic redistribution within the active layer. The resulting current transients were recorded using a Keithley 2400 source meter and a custom LabView interface. The extracted

charge was calculated by integrating the current transient, and the charge carrier density was determined by dividing the total charge by the elementary charge and device volume.

Dynamic PL(V) Measurements: Voltage-dependent photoluminescence (PL) measurements were conducted by illuminating a defined pixel area of the perovskite solar cell with a continuous-wave (CW) 520 nm laser. An external voltage bias (e.g., 0 V or open-circuit voltage, Voc) was applied using a Keithley 2400 SourceMeter during illumination. To ensure accurate detection from the active region only, the devices were precisely masked to eliminate PL signals from non-active areas. Time-resolved PL spectra were recorded using an Andor Solis system equipped with a high-sensitivity silicon detector, enabling detailed spectral monitoring throughout the experiment. Each measurement condition was repeated to confirm reproducibility, demonstrating consistent and stable PL responses under different applied biases.

XPS measurement: XPS measurements were conducted using a JEOL JPS-9030 setup with a base pressure of 2 · 10−9 mbar. A hemispherical analyzer with pass energy of 20 eV was used to detect the emitted photoelectrons, yielding a resolution of 1.0–1.1 eV. The binding energy scale of the analyzer was calibrated by setting the Au 4f7/2and the Cu 2p3/2 peaks of clean gold and copper foils to 84.0 and 932.6 eV, respectively.

Long Term PL Stability Measurements: Alustar G2 LED from ledxon GmbH was used to illuminate the film under investigation. A PS-1302 D power supply from VOLTCRAFT was used as the power supply for LED. The PL of the film was recorded by OCEAN SR compact spectrometer. The PL of the sample was recorded every minute under continues illumination. The measurement took place in the ambient air with encapsulated samples.

Maximum power point (MPP) tracking measurement: $CsMAFAPbI_3$ and CsMAFA(PbSn)I3 were measured under continues 1sun ($100mW/cm^2$) illumination, while $FASnI_3$, and DMSO-free $FASnI_3$ samples were tested under 1sun (AM 1.5G) illumination with UV380 filter. Unencapsulated $FASnI_3$, and DMSO-free $FASnI_3$ devices were measured in the Aging ring filled with N2 atmosphere (at room tempreture) whereas $CsMAFA(PbSn)I_3$ was measured (at room tempreture) in N_2 filled glovebox. $CsMAFAPbI_3$ device was encapsulated and measured in the ambient air. The initial set temperature for the $CsMAFAPbI_3$ device was room temperature, although the temperature could rise up to ~37 °C during operation due to instrumental heating.

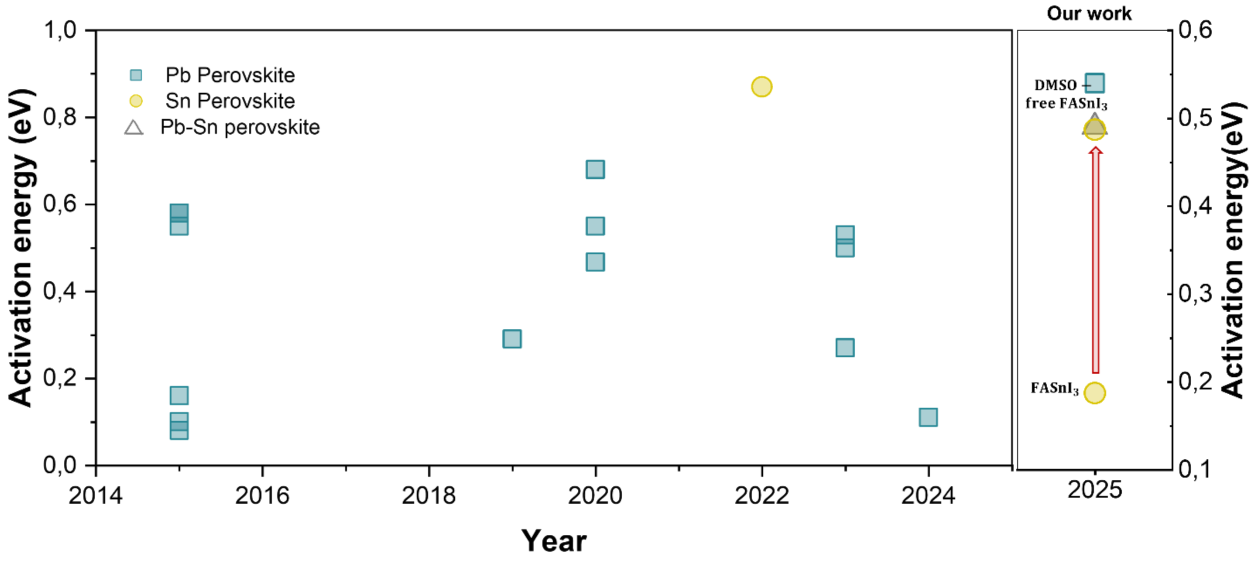

Figure S 1. Reported activation energies for Pb and Sn perovskites.[1–12] *$FASnI_3$ solar cell shows relatively smaller activation energy, while elimination DMSO results in higher migration barrier for ion migration in DMSO free $FASnI_3$.*

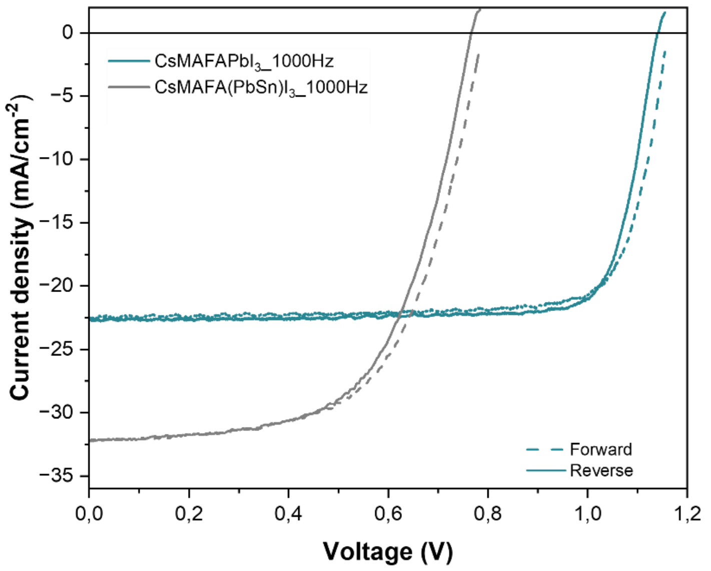

Figure S2. J-V characteristics of $CsMAFAPbI_3$ and $CsMAFA(PbSn)I_3$ at 1000Hz. *Both devices show inverted hysteresis at 1000Hz.*

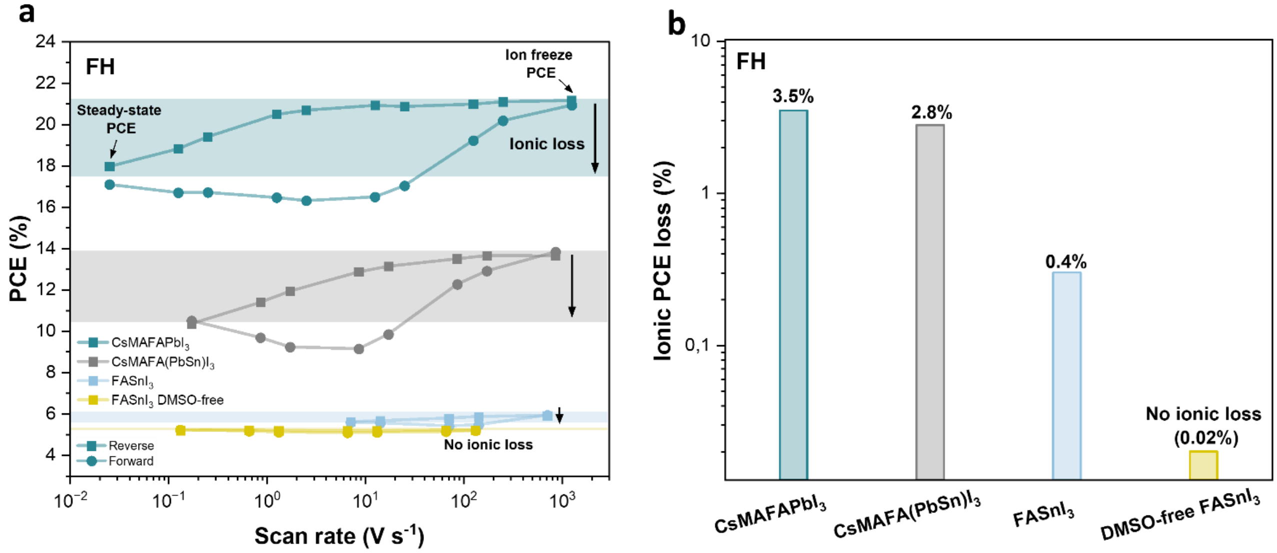

Figure S3. PCEs adapted from FH measurement for Pb and Sn-based perovskite solar cell and calculated ionic PCE loss. *The DMSO-free $FASnI_3$ device exhibits no ionic loss.*

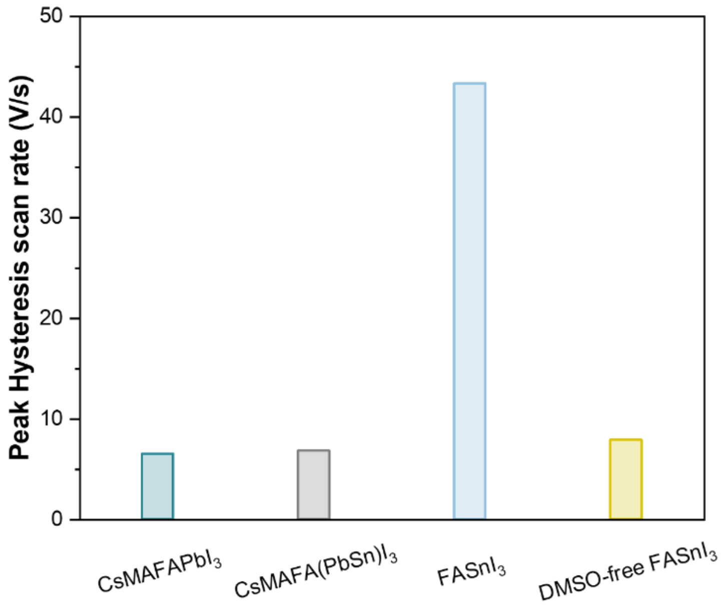

Figure S4. Peak hysteresis scan rate extracted from fast hysteresis measurement

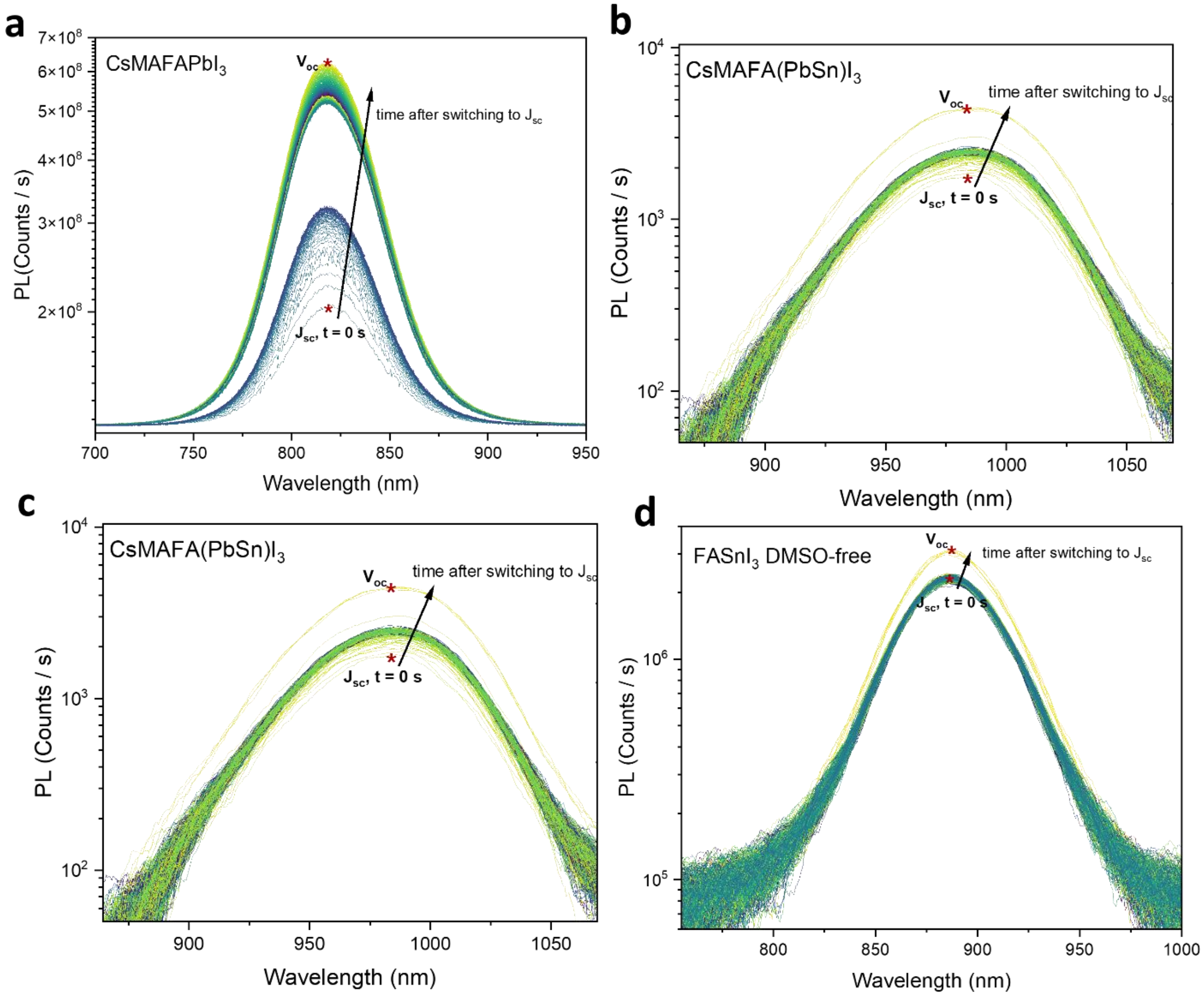

Figure S5. a-d) Voltage-dependent photoluminescent measurement on different perovskite solar cells.

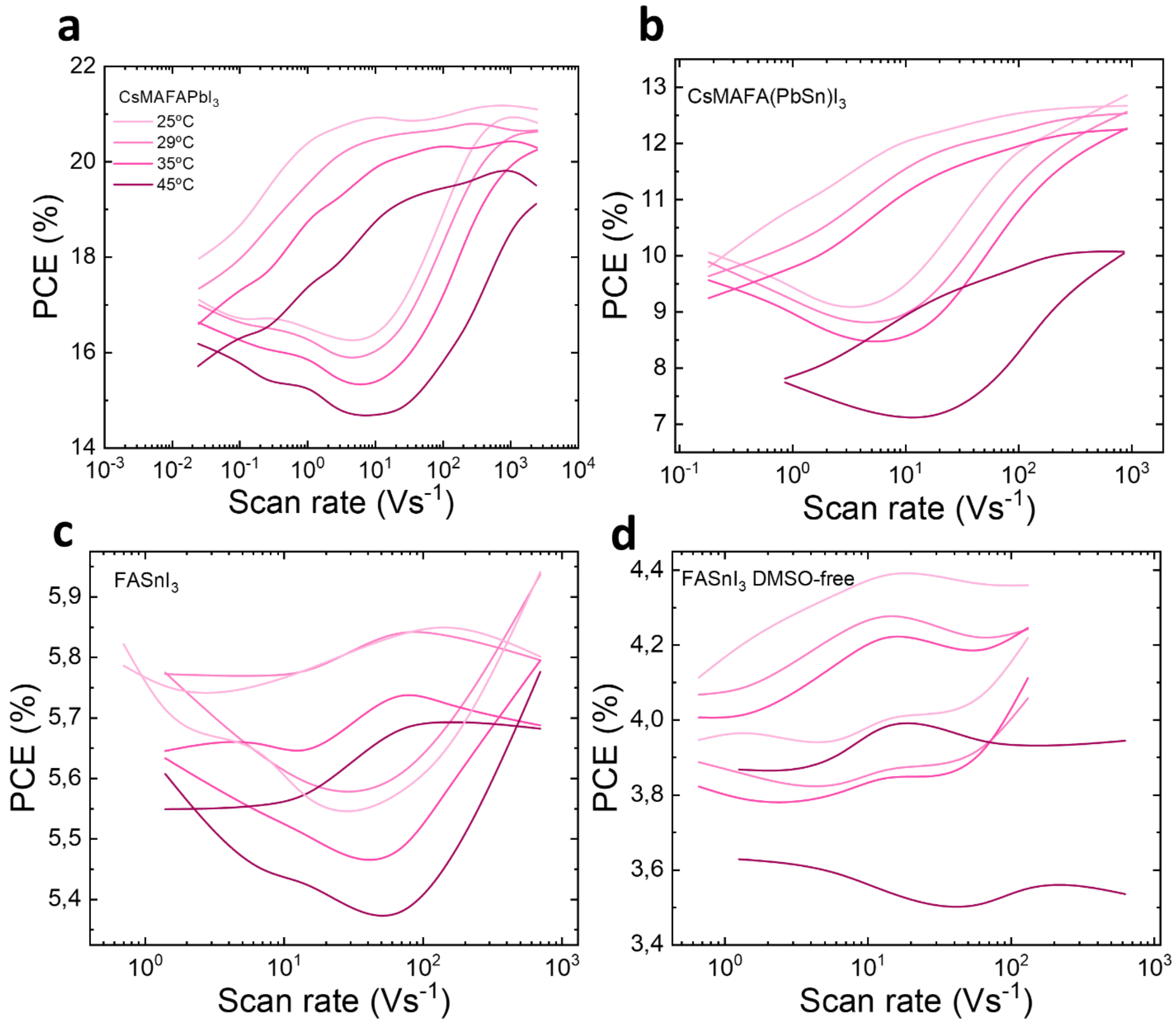

Figure S6. Interpolated PCE values adopted from Temperature dependent fast hysteresis for various perovskite solar cells at 25, 29, 35, 45 °C.

Figure S7 represents the XPS of the ITO/PEDOT: PSS/ DMSO-free and DMSO processed $FASnI_3$ perovskite. The Sn^{4+} in DMSO-free sample (6.5%) is a slightly lower than that in DMSO sample (7.7%), indicating a reduced oxidation in DMSO-free solvent system. We believe that the remaining Sn^{4+} content in both DMSO and DMSO-free samples could be attributed to the oxidation due to acidity of PEDOT: PSS.

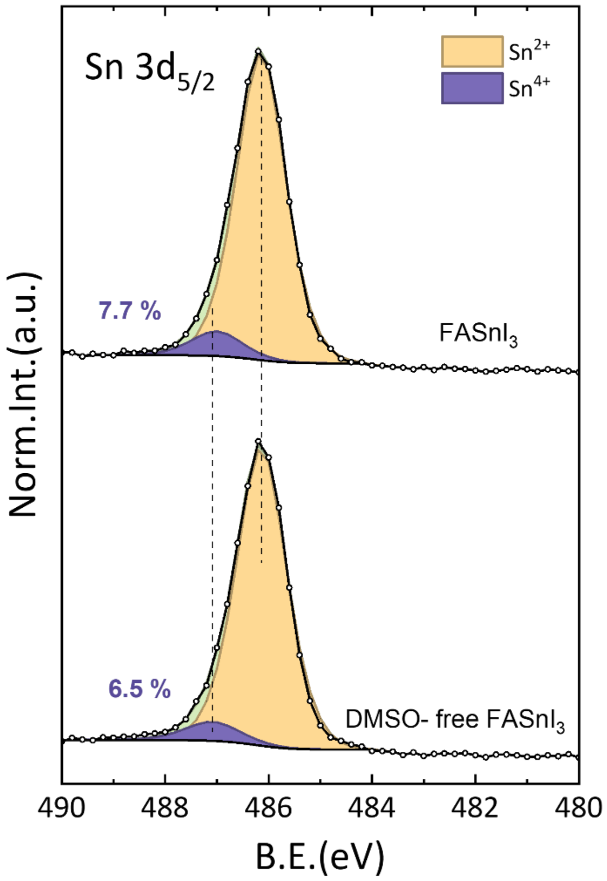

Figure S7. XPS of the ITO/PEDOT: PSS/ DMSO-free and DMSO processed $FASnI_3$ perovskite

References

(1) Azpiroz, J. M.; Mosconi, E.; Bisquert, J.; De Angelis, F. Defect Migration in Methylammonium Lead Iodide and Its Role in Perovskite Solar Cell Operation. *Energy Environ Sci* **2015**, *8* (7), 2118–2127. https://doi.org/10.1039/c5ee01265a.

(2) Haruyama, J.; Sodeyama, K.; Han, L.; Tateyama, Y. First-Principles Study of Ion Diffusion in Perovskite Solar Cell Sensitizers. *J Am Chem Soc* **2015**, *137* (32), 10048–10051. https://doi.org/10.1021/jacs.5b03615.

(3) Tan, S.; Yavuz, I.; De Marco, N.; Huang, T.; Lee, S. J.; Choi, C. S.; Wang, M.; Nuryyeva, S.; Wang, R.; Zhao, Y.; Wang, H. C.; Han, T. H.; Dunn, B.; Huang, Y.; Lee, J. W.; Yang, Y. Steric Impediment of Ion Migration Contributes to Improved Operational Stability of Perovskite Solar Cells. *Advanced Materials* **2020**, *32* (11). https://doi.org/10.1002/adma.201906995. 1906995- 1906995

(4) Diekmann, J.; Peña-Camargo, F.; Tokmoldin, N.; Thiesbrummel, J.; Warby, J.; Gutierrez-Partida, E.; Shah, S.; Neher, D.; Stolterfoht, M. Determination of Mobile Ion Densities in Halide Perovskites via Low-Frequency Capacitance and Charge Extraction Techniques. *Journal of Physical Chemistry Letters* **2023**, *14* (18), 4200–4210. https://doi.org/10.1021/acs.jpclett.3c00530.

(5) Eames, C.; Frost, J. M.; Barnes, P. R. F.; O'Regan, B. C.; Walsh, A.; Islam, M. S. Ionic Transport in Hybrid Lead Iodide Perovskite Solar Cells. *Nat Commun* **2015**, *6*. https://doi.org/10.1038/ncomms8497.

(6) Azpiroz, J. M.; Mosconi, E.; Bisquert, J.; De Angelis, F. Defect Migration in Methylammonium Lead Iodide and Its Role in Perovskite Solar Cell Operation. *Energy Environ Sci* **2015**, *8* (7), 2118–2127. https://doi.org/10.1039/c5ee01265a.

(7) Futscher, M. H.; Lee, J. M.; McGovern, L.; Muscarella, L. A.; Wang, T.; Haider, M. I.; Fakharuddin, A.; Schmidt-Mende, L.; Ehrler, B. Quantification of Ion Migration in CH3NH3PbI3 Perovskite Solar Cells by Transient Capacitance Measurements. *Mater Horiz* **2019**, *6* (7), 1497–1503. https://doi.org/10.1039/c9mh00445a.

(8) Reichert, S.; An, Q.; Woo, Y. W.; Walsh, A.; Vaynzof, Y.; Deibel, C. Probing the Ionic Defect Landscape in Halide Perovskite Solar Cells. *Nat Commun* **2020**, *11* (1). https://doi.org/10.1038/s41467-020-19769-8.

(9) Qu, S.; Huang, H.; Wang, J.; Cui, P.; Li, Y.; Wang, M.; Li, L.; Yang, F.; Sun, C.; Zhang, Q.; Zhu, P.; Wang, Y.; Li, M. Revealing and Inhibiting the Facet-Related Ion Migration for Efficient and Stable Perovskite Solar Cells. *Angewandte Chemie - International Edition* **2025**, *64* (4). https://doi.org/10.1002/anie.202415949.

(10) Zhang, J.; Niu, X.; Peng, C.; Jiang, H.; Yu, L.; Zhou, H.; Zhou, Z. Inhibiting Ion Migration Through Chemical Polymerization and Chemical Chelation Toward Stable Perovskite Solar Cells. *Angewandte Chemie - International Edition* **2023**, *62* (50). https://doi.org/10.1002/anie.202314106.

(11) Chen, C.; Song, Z.; Xiao, C.; Awni, R. A.; Yao, C.; Shrestha, N.; Li, C.; Bista, S. S.; Zhang, Y.; Chen, L.; Ellingson, R. J.; Jiang, C. S.; Al-Jassim, M.; Fang, G.; Yan, Y. Arylammonium-Assisted Reduction of the Open-Circuit Voltage Deficit in Wide-Bandgap Perovskite Solar Cells: The Role of Suppressed Ion Migration. *ACS Energy Lett* **2020**, *5* (8), 2560–2568. https://doi.org/10.1021/acsenergylett.0c01350.

(12) Tayagaki, T.; Yamamoto, K.; Murakami, T. N.; Yoshita, M. Temperature-Dependent Ion Migration and Mobile-Ion-Induced Degradation of Perovskite Solar Cells under Illumination. *Solar Energy Materials and Solar Cells* **2023**, *257*. https://doi.org/10.1016/j.solmat.2023.112387.
